# Advances in the Study of Extracellular Vesicles for Bone Regeneration

**DOI:** 10.3390/ijms25063480

**Published:** 2024-03-20

**Authors:** Yao Jiao, Yitong Liu, Juan Du, Junji Xu, Zhenhua Luo, Yi Liu, Lijia Guo

**Affiliations:** 1 Department of Periodontics, School of Stomatology, Capital Medical University, Beijing 100050, China; jiaoyao716@163.com (Y.J.); 15010546495@163.com (Y.L.); dujuan_1983@163.com (J.D.); uujkl@163.com (J.X.); laryhua@163.com (Z.L.); 2 Laboratory of Tissue Regeneration and Immunology and Department of Periodontics, Beijing Key Laboratory of Tooth Regeneration and Function Reconstruction, School of Stomatology, Capital Medical University, Beijing 100050, China; 3 Department of Orthodontics (WangFuJing Campus), School of Stomatology, Capital Medical University, Beijing 100006, China

**Keywords:** extracellular vesicles, bone regeneration, exosomes, bone repair

## Abstract

Promoting the efficiency of bone regeneration in bone loss diseases is a significant clinical challenge. Traditional therapies often fail to achieve better therapeutic outcomes and shorter treatment times. However, in recent years, extracellular vesicles (EVs) have gained significant attention due to their exceptional osteogenic function in bone regeneration and superior therapeutic effects compared to traditional cell therapy. EVs have emerged as a promising therapy for tissue defect regeneration due to their various physiological functions, such as regulating the immune response and promoting tissue repair and regeneration. Moreover, EVs have good biocompatibility, low immunogenicity, and long-term stability, and can be improved through pretreatment and other methods. Studies investigating the mechanisms by which extracellular vesicles promote bone regeneration and applying EVs from different sources using various methods to animal models of bone defects have increased. Therefore, this paper reviews the types of EVs used for bone regeneration, their sources, roles, delivery pathways, scaffold biomaterials, and applications.

## 1. Introduction

Bone defects are intricate pathological alterations resulting from osteoporotic fractures, traumatic injuries, inflammatory responses, malignant tumors, and various other factors. Globally, osteoporosis-related fractures occur at a rate of one every 20 s among individuals aged 50 and above, with over 2 million bone graft procedures conducted annually [1]. The extended healing duration associated with traditional treatments contributes significantly to the substantial healthcare expenses incurred and demonstrates certain inherent limitations [2]. The primary traditional treatment for bone defects is bone grafting, which can involve autologous bone, allogeneic bone, or synthetic materials [3]. However, autologous bone graft treatment has several drawbacks, including poor bone volume, limited availability, donor site damage, and other complications [4,5]. Conversely, allogeneic transplants can increase the risk of disease transmission, angiogenesis problems, immune rejection, and other issues [6]. To address these challenges, sustainable bone regeneration therapies are emerging, such as scaffolds, bioactive substances, and cells or tissues with osteogenic potential [7]. There are also inevitable challenges associated with cell therapy, such as biological safety concerns, limited tissue sources, and ethical issues. Additionally, the ischemic microenvironment of bone injuries may lead to a reduced survival rate of transplanted cells, making it difficult to ensure efficacy. Therefore, the emergence of cell-free therapies provides a new opportunity for bone regeneration treatment. Extracellular vesicles (EVs) can induce osteogenesis, angiogenesis, and regulate immunity. They contain fewer membrane proteins, making clinical applications safer and with a higher yield. As such, EVs are expected to be an ideal component to combine with bone engineering scaffolds to guide bone regeneration [8,9].

**Figure 1:**
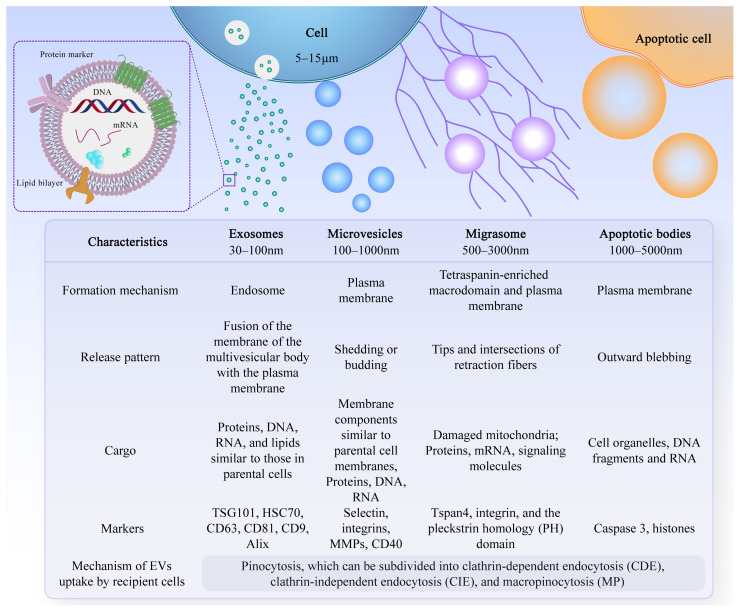
Characteristics of different types of EVs. There are different types of EVs, and their formation mechanisms, release patterns, cargo, and markers are not exactly the same.

Extracellular vesicles are small lipid bilayer membrane particles secreted by all cell types. The term “EVs” collectively refers to diverse vesicle types, such as exosomes, microvesicles, microparticles, shedding vesicles, and apoptotic bodies (Figure 1). These heterogeneous families of small vesicles are conventionally classified into the following three groups, according to their size and biogenesis: exosomes (30–100 nm), microvesicles (100–1000 nm), and apoptotic bodies (1000–5000 nm) [10]. Their contents include DNA fragments, messenger ribonucleic acids (mRNAs), proteins, and lipids [11,12]. Exosomes are formed within multivesicular bodies and are released when these bodies fuse with the plasma membrane. They contain proteins and lipids derived from the parent cells, including tetraspanin (CD9, CD63, and CD81), proteins involved in multivesicular body biosynthesis [such as Alix and tumor susceptibility gene 101, (TSG101)], heat shock proteins (HSP70 and HSP90), and membrane translocation and fusion proteins (GTPases and membrane coupling proteins) [13]. Microvesicles are produced and released by budding from the plasma membrane. Apoptotic bodies are vesicles formed during apoptosis that contain nuclear and cytoplasmic fragments surrounded by membranes when cells shrink and break apart. In recent years, researchers have discovered a new type of extracellular vesicle, called a migrasome, which is a large vesicle growing at the tip or crossing of retraction fibers in the back of migrating cells. It is about 500 nm to 3000 nm in diameter and contains numerous smaller vesicles [14]. After the cells migrate, the retraction fibers eventually break, releasing the migrasomes into the extracellular space. Compared with exosomes, migrasomes have specific proteins, such as N-Deacetylase/N-Sulfotransferase 1 (NDST1), EGF domain-specific O-linked N-acetylglucosamine transferase (EOGT), Phosphatidylinositol glycan anchor biosynthesis class K (PIGK), and Carboxypeptidase Q (CPQ) [15]. A recent study has shown that migrasomes promote angiogenesis in chick embryos [16]. However, there have been no studies on the use of migrasomes for the treatment of specific diseases.

After leaving the initiating cell, these vesicles can reach the target cell via markers on their membrane surface, which can interact with the receptor-ligand, and thus alter the physiological state of the target cell by transferring their contents or triggering signals on the target cell’s surface. The effective uptake of EVs by cells is crucial for their biological activity. However, the precise mechanism underlying the uptake of EVs by recipient cells remains incompletely understood. Recent research suggests that the uptake mechanism primarily involves pinocytosis, which can be categorized into clathrin-dependent endocytosis (CDE), clathrin-independent endocytosis (CIE), and macropinocytosis (MP), among which CIE and MP are the most common modalities [17,18,19,20]. Increasingly, studies have shown that EVs have multiple physiological functions, such as regulating the body’s immune response, promoting tissue regeneration and repair, and neural communication [21]. Due to their excellent biocompatibility, long-term stability, and low immunogenicity, EVs have attracted widespread exploration and application, especially in the field of bone regeneration [22]. In this review, we discuss the related knowledge and research progress of EVs promoting bone regeneration, including the sources of EVs for the treatment of bone regeneration, as well as their functions and applications.

## 2. Common Sources of EVs for Bone Regeneration

### 2.1. Immune Cells

**Figure 2:**
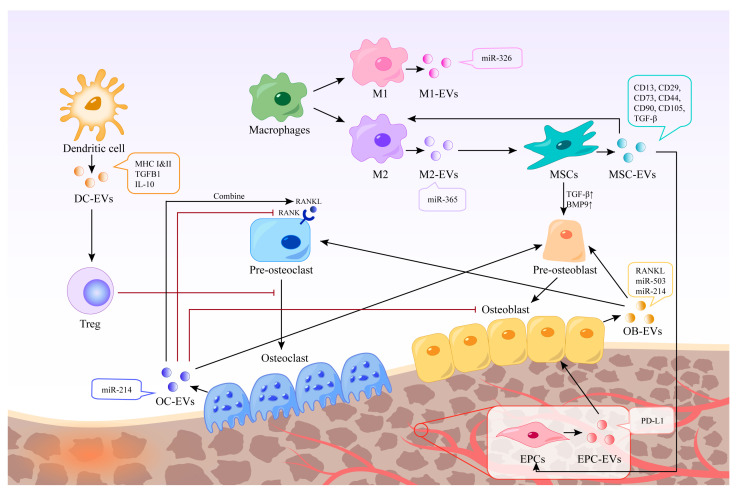
Different EVs in bone regeneration. EVs derived from various cells possess distinct contents and functions. Certain EVs can act directly on bone cells, whereas others can indirectly stimulate bone regeneration by regulating immune cells, inhibiting osteoclasts, and promoting endothelial cell regeneration. The black arrow represents the process of EVs production and action, and the red arrow represents the inhibitory effect of EVs or cells.

Immune cells in the bone microenvironment release cytokines and paracrine factors that exhibit activating or inhibitory responses to bone-associated cells. Neutrophils are the most abundant white blood cells in the blood circulation, and they are also one of the first types of immune cells recruited in the microenvironment of bone injury and inflammatory response. Studies have shown that Thrombospondin-1 (TSP-1), an acellular glycoprotein associated with blood clot formation and angiogenesis, is strongly expressed in response to the stimulation of neutrophil-derived exosomes [23]. TSP-1 can trigger CD36-dependent signal that reduces the sensitivity of platelets to PGE-1 stimulated by endothelium-derived mediators, thereby impairing their ability to inhibit platelets [24]. Mast cells are widely distributed around microvessels in the skin and visceral submucosa, which promote the secretion of coagulation factors in the inflammatory process and participate in immune regulation. When activated, mast cell-derived exosomes can activate endothelial cells to secrete plasminogen activator inhibitor type 1 (PAI-1) [25]. Dendritic cells (DCs) can regulate the initiation of adaptive immunity by secreting EVs containing major histocompatibility complex (MHC) class I and II molecules to activate cognate T cells and promote humoral responses. Studies have shown that dendritic cell-derived EVs can induce osteogenesis [26]. Their exosomes contain immunomodulators, such as transforming growth factor-beta 1 (TGF-β1) and interleukin-10 (IL-10), which can be released in response to inflammation, promoting the recruitment of regulatory T cells to inhibit osteoclasts and reduce bone loss [27]. Macrophages are a ubiquitous cell type in vertebrate tissues, serving as a primary defense against pathogens by phagocytosing microorganisms, infected particles, and dead cells [28]. Their differentiation into M1 or M2 phenotypes is modulated by the local environment, with exosomes derived from macrophages reflecting their respective phenotypic characteristics [29] (Figure 2). These exosomes contain distinct biological information, resulting in unique functions; for instance, M2-Exos have been shown to contain higher levels of miR-365, whereas miR-326 is more abundant in M1-Exos [30,31]. Notably, no biomarkers have been identified to distinguish M1-Exos from M2-Exos [32]. In a study aimed at promoting osteogenesis, Chen et al. combined M2 macrophage-derived exosomes and stromal cell-derived factor-1α (SDF-1α) with hydrogels, yielding a hydrogel with good biocompatibility, hemostatic ability, and healing promotion. In vitro experiments revealed that the hydrogel could facilitate the proliferation and migration of human bone marrow mesenchymal stem cells and human umbilical vein endothelial cells, ultimately promoting osteogenesis and angiogenesis [33].

### 2.2. Stem Cells

Mesenchymal stem cells (MSCs) are multipotent stromal cells with various sources, such as bone marrow-derived mesenchymal stem cells (BMSCs), adipose-derived mesenchymal stem cells (ASCs), umbilical cord-derived mesenchymal stem cells (UMSCs), and others [34,35,36]. BMSCs have been widely used in bone regeneration strategies due to their osteogenic capacity [37]. EVs derived from stem cells have been shown to have stem cell-like regenerative functions. Thus, using EVs instead of stem cells to treat tissue defects can avoid the side effects of stem cell therapy, such as immune response and tumor formation [38]. EVs are also easier to store and transport. In addition to common surface markers such as CD9 and CD81, exosomes derived from MSCs also express CD73, CD44, and CD90, which are characteristic markers of MSCs [39]. Characterization of BMSC-derived exosome contents based on proteomics identified 730 functional proteins, including proteins that control the growth, proliferation, adhesion, migration, and morphogenesis capacities of MSCs [40]. These extracellular vesicles can promote the expression of osteogenic growth factors and bone-related proteins, and increase calcium deposition and matrix mineralization in vitro [41,42,43]. BMSC-derived EVs showed characteristic markers CD13, CD29, CD44, CD73, CD90, and CD105 [44], which can up-regulate the expression of TGF-β1 and bone morphogenetic protein 9 (BMP9), thereby promoting the differentiation of osteoblasts [45]. Qin et al. isolated BMSC-derived EVs and found that they positively regulate osteogenic genes and osteoblast differentiation in vitro. In vivo, experiments using rats with skull defects showed that EVs lead to more bone formation in bone defects, and miR-196a may play a crucial role [46].

### 2.3. Bone Cells

Bone homeostasis is regulated by interactions among osteoblasts, osteocytes, and osteoclasts and their surrounding microenvironment [47]. Exosomes from bone cells, immune cells, mesenchymal stem cells, and endothelial cells have been shown to affect bone formation and resorption, potentially influencing the development of bone-related diseases [48]. Osteoclasts are multinucleated cells derived from bone marrow monocytes and macrophages responsible for bone resorption. EVs derived from mature osteoclasts contain competitive inhibitors of receptor activator of nuclear factor kappa-Β (NF-κB), which inhibit osteoclast generation in the same environment [49]. Moreover, EVs released by mature osteoclasts can bind to receptor activator of nuclear factor-kappa B ligand (RANKL) on the surface of osteoblasts and trigger the RANKL reverse signaling pathway, thereby activating the key Runt-related transcription factor 2 (Runx2) and promoting bone formation [50]. Osteoclast-derived exosomes have been shown to promote osteogenic differentiation of stromal cells before osteogenesis [51]. However, it has also been shown to inhibit their differentiation and lead to reduced bone formation by being internalized in osteoblasts through EphrinA2/EphA2 recognition [52]. Li et al. found that miR-214-3p levels in osteoclasts were elevated in ovariectomized mice and elderly women with fractures, and that miR-214-3p in osteoclast-derived EVs was able to transfer to osteoblasts in vitro to inhibit osteoblast activity and reduce bone formation in vivo [53]. Osteoblasts are resident bone cells derived from bone marrow mesenchymal stem cells and are responsible for bone matrix synthesis and mineralization by releasing collagen and glycoproteins. Mineralized osteoblast-derived exosomes have been shown to induce osteogenic differentiation through activation of the Wnt signaling pathway, calcium signaling, and regulation of microRNA profiling [54]. Meanwhile, osteoblast-derived exosomes are also rich in RANKL protein, which can stimulate osteoclast differentiation through the RANKL-RANK signaling pathway and lead to nuclear translocation of nuclear factor of activated T cells, cytoplasmic 1 (NFATc1), a major transcriptional regulator of osteoclast differentiation [55]. In contrast, another study showed that mineralized osteoblasts were able to release EVs containing miR-503-3p, which impaired osteogenesis by inhibiting RANK expression [56]. This may be due to the heterogeneity of EVs, and the mechanisms regulating the switch between bone formation and bone resorption are not fully understood [57].

### 2.4. Endothelial Cells

Angiogenesis plays a crucial role in the bone regeneration microenvironment. Exosomes derived from endothelial cells can target osteocytes and stimulate bone regeneration [58]. Studies have demonstrated that exosomes derived from endothelial progenitor cells (EPCs) can promote angiogenesis through the Raf/ERK signaling pathway, thereby accelerating bone formation [59]. Moreover, EPCs were found to enhance healing and neovascularization in a mouse fracture model by recruiting osteoclast precursors. EPC-derived exosomes have also been shown to have a positive impact in animal models of osteoporosis, mainly through the high ferritin pathway in osteoblasts [60].

## 3. Functions of EVs

**Figure 3:**
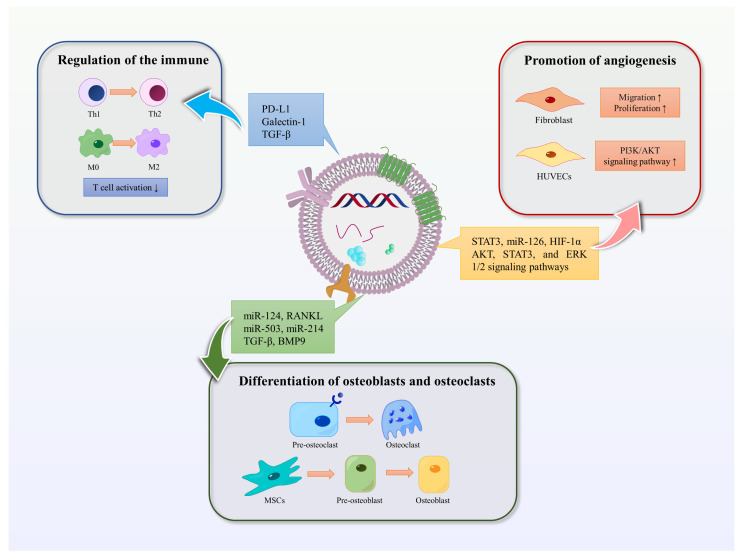
Functions of EVs. The functions of EVs in promoting bone regeneration include regulating immunity, promoting angiogenesis, and promoting osteoblast and osteoclast differentiation. Blue arrows represent the immunomodulatory effect of EVs in bone regeneration, red represents the promotion of angiogenesis, and green represents the effect on osteoblasts or osteoclasts.

EVs are involved in promoting bone regeneration in various ways, including regulation of the immune environment, promotion of angiogenesis, differentiation of osteoblasts and osteoclasts, and promotion of bone mineralization (Figure 3).

### 3.1. Mediating Immune Stimulation or Immunosuppression

Moderate inflammatory response is necessary in the early stage of bone injury, while hyperactive and persistent inflammation can hinder bone regeneration and lead to inflammatory injury. EVs have the potential to act as immunomodulatory messengers by mediating immune stimulation or immunosuppression [61]. MSC-derived exosomes can influence the activity of immune cells, including T cells, B cells, NK cells, and macrophages. A clinical study has shown that MSC-derived exosomes may reduce the ability of peripheral blood mononuclear cells (PBMCs) to release proinflammatory cytokines in vivo. MSC-derived exosomes upregulate IL-10 and TGF-β1 in PBMCs, thereby promoting the proliferation and immunosuppressive capacity of Tregs to reduce inflammatory damage [62]. In addition, human umbilical vein endothelial cells (HUVECs) -derived exosomes contain a high concentration of programmed death ligand-1 (PD-L1). Exosomes overexpressing PD-L1 can specifically bind to programmed death-1 (PD-1) on T cells, inhibit the activation of T cells, and promote callus formation and fracture healing [63]. Studies have shown that mesenchymal cell-derived microvesicles (MVs) can deliver several immunomodulators such as PD-L1, galectin-1, and TGF-β, which can inhibit self-reactive cells and suppress their mediated tissue damage, induce peripheral tolerance, and modulate immune responses [64]. Furthermore, MSC-derived exosomes have been found to inhibit the concentrations of pro-inflammatory cytokines such as interleukin-1 beta (IL-1β) and tumor necrosis factor-alpha (TNF-α), while the secretion of TGF-β is increased. This induces the conversion of Th1 to Th2 cells and inhibits the pro-inflammatory response, thereby reducing inflammation and promoting anti-inflammatory response in a similar manner to MSCs [65]. In addition, M2-type macrophages have an anti-inflammatory phenotype and are mainly responsible for tissue remodeling during macrophage polarization. Studies have shown that MSC-derived microvesicles can promote the polarization of monocytes to M2-type macrophages, thereby mediating tissue repair [66].

### 3.2. Promotion of Angiogenesis

EVs can also play a crucial role in promoting angiogenesis. In vitro studies have shown that BMSC-Exos can promote fibroblast migration and proliferation through signaling pathways involving AKT, STAT3, and ERK 1/2 [67]. Furthermore, BMSCs are enriched in transcriptionally active STAT3, a transcription factor that is involved in angiogenesis, proliferation, migration, and growth factor production. iPS-MSC-Exos, which are secreted by induced pluripotent stem cell-derived mesenchymal stem cells, have been shown to have great potential in treating ischemic tissues. Liu et al. found that after intravenous injection into a rat model of steroid-induced osteonecrosis, iPS-MSC-Exos significantly prevented bone loss and promoted angiogenesis in the femur [68]. Hypoxic preconditioning can enhance the regenerative capacity of stem cells. Ding et al. reported that miR-126 was significantly upregulated in BMSC-Exos under hypoxic conditions compared to the normal group. MiR-126, which is involved in the process of angiogenesis, can induce the activation of the PI3K/AKT pathway in HUVECs, thereby promoting the formation of new blood vessels [69]. Moreover, research has shown that transplantation of umbilical cord MSC-derived exosomes (uMSC-Exos) combined with hydrogel into the site of injury in a rat model of femur fracture resulted in uMSC-Exos promoting bone healing through hypoxia-inducible factor 1α (HIF-1α)-mediated pro-angiogenic effects [70].

### 3.3. Differentiation of Osteoblasts and Osteoclasts

EVs play a role in promoting the differentiation of bone marrow mesenchymal stem cells into osteoblasts and osteoclasts, thus maintaining the balance of bone metabolism [71]. Paracrine signaling mediated by EVs regulates bone homeostasis by affecting osteoblasts and osteoclasts [72]. Furthermore, miR-214-3p in osteoblast-derived exosomes can be transferred to osteoblasts, inhibiting osteoblast activity in vitro and reducing bone formation in vivo [53]. Annexins and sodium-dependent inorganic phosphate transporters transport calcium and phosphate for the initial formation and accumulation of hydroxyapatite crystals in matrix vesicles. These vesicles later release these crystals into the extracellular fluid inducing calcification following collagen calcification [73,74].

## 4. Application of EVs in Bone Regeneration

### 4.1. Isolation, Storage, and Management of EVs

Purity is critical before applying EVs to the clinic, and isolation and purification techniques should be standardized. Differential centrifugation (DC) is one of the most commonly used separation methods, but the separation efficiency is low and time-consuming [75]. Tangential flow filtration (TFF) has been widely used in industry due to its advantages of high efficiency, flexibility, and scalability [76]. Whereas size cannot be used as the sole criterion to distinguish vesicle types, TFF may allow similarly sized contaminants or nonvesicular particles to flow together. Therefore, TFF can be used in combination with other techniques, such as the immunomagnetic bead capture method, density gradient ultracentrifugation (DG UC), anion exchange chromatography (AEC), and various principles of microfluidics systems, which is the current trend for the isolation of EVs [77]. In addition, the stability of EVs is very important in trials or clinical applications, but it is often easily overlooked. Although the bilayer structure makes exosomes resistant to degradation to a certain extent, EVs are unstable. Temperature is the decisive factor for EVs storage. Studies have shown that it can be maintained at room temperature for less than 48 h [78]. At the storage temperature of 4 °C, the number and antibacterial ability of EVs is observed to decrease in a short time, which may be related to their aggregation, fusion, adsorption to the tube wall, and decomposition [79]. Storage at −20 °C affects the size of EVs, while the total number is relatively stable, a temperature of −80 °C seems to be most suitable for long-term storage [80]. In addition to temperature, more and more people are investigating methods to improve its stability. The slow degradation rate of hydrogel-encapsulated exosomes is an effective method. Liu et al. used nanocomposite hydrogel as a carrier of exosomes to extend the time of BMSC-derived exosomes in the periodontal pocket and enhance their osteogenic function [81].

### 4.2. Delivery Method of EVs

In bone regeneration engineering, EVs play a crucial role in promoting tissue regeneration and can be delivered to the designated injury site through a variety of methods. Currently, EVs are utilized as a biologic agent to treat tissue damage by both in vivo and topical injection. Osteoarthritis is mainly managed through pain medication, with no satisfactory treatment available to improve joint stability [82]. However, extracellular vesicles have been utilized in the exploration of osteoarthritis treatment, given their ability to promote bone tissue regeneration. Exosomes extracted from bone marrow-derived mesenchymal cells were injected into the joints of mice with osteoarthritis, and BMMSC-Exos were found to be effective in treating osteoarthritis, as evidenced by a significant increase in type II collagen expression [83]. Injection therapy has limitations in achieving sustained aggregation and controlled release due to immune system clearance in the body, thus weakening their function [84]. Direct treatment of EVs cannot reach the appropriate concentration required for treatment, and off-target effects and accumulation in non-target organs are inevitable [85,86]. Moreover, local EVs injection therapy is inefficient in the treatment of large bone defects, and its mechanical supporting capacity and carrying capacity are insufficient. To overcome these limitations, many studies have explored the combination of biological materials with EVs as loading carriers.

Scaffold materials commonly used for bone regeneration include synthetic polymers, natural polymers, and bioceramics. Examples of commonly used synthetic polymers are polycaprolactone, polypropylene glycolate, and polyglycolic acid [87,88]. Loading and delivering EVs from biomaterials are promising tools for bone regeneration. In recent years, researchers have experimented with new biological scaffolds as slow-release carriers for EVs to maintain their biological activity and retention time at the site of bone defects, which can further accelerate the efficiency and effectiveness of bone regeneration. EVs can bind to biological scaffolds and provide a safe and stable carrier for the in vivo delivery of EVs. Different composite technologies have been applied in the loading process of EVs, including physical adsorption [35], chemical cross-linking [89], specific binding [90], lyophilization [91], 3D printing [92], and more. Some studies have shown that combining EVs with biological scaffolds has a better effect on repairing bone defects than using EVs alone [93]. For example, Wu et al. compared the therapeutic effects of β-tricalcium phosphate (β-TCP) alone and SHED-derived exosomes combined with β-TCP in a rat model of periodontal injury. The exosomes/β-TCP group exhibited better bone regeneration than either the β-TCP group or the control group, and SHED-derived exosomes were found to promote periodontal bone regeneration through the AMPK signaling pathway by promoting new angiogenesis and osteogenesis [94]. Additionally, hASC-derived exosomes were combined with polydopamine-coating PLGA (PLGA/pDA) scaffolds for the treatment of cranio-parietal defects in mice, which showed slow and sustained release of exosomes from PLGA/pDA scaffolds. This combination significantly enhanced bone regeneration by promoting the migration and homing ability of MSCs around the defect [95]. EVs can also enhance the mechanical strength of biological materials. For instance, Qayoom et al. used a calcium sulfate/nanohydroxyapatite-based nanocement (NC) as a carrier of MSC-derived exosomes, which improved the biomechanical strength and promoted bone tissue formation in the femoral neck canal defect of osteoporotic rats [96]. However, the efficacy of EVs combined with biological scaffolds may be influenced by factors such as stress distribution at the damaged site, degradation rate of biological materials, and material properties. Therefore, numerous in vivo studies are necessary to elucidate the standard and process of EVs treatment for bone regeneration, enabling it to be better applied in clinical treatments.

### 4.3. Application Direction

EVs are now widely used in the treatment of bone defects. In addition to the above-mentioned applications, which promote fracture healing, bone defect healing, and the treatment of osteoporosis and osteoarthritis, EVs can also be utilized in the treatment of periodontal tissue loss. Periodontitis is a chronic inflammatory disease, and is one of the most common chronic infections in humans. It leads to the destruction of periodontal tissues, including alveolar bone, periodontal ligaments, and cementum [97]. Currently, there is no effective treatment available to repair inflammatory bone loss in periodontitis. In contrast, extracellular vesicles offer a promising new avenue for treating periodontitis and improving alveolar bone resorption [98]. Although MSC-derived exosomes have demonstrated therapeutic potential in experimental periodontitis, their clinical application is hindered by their low yield and limited efficacy. To address this issue, Zhang et al. used 3D systemic culture to extract exosomes, and found that they were able to exert enhanced anti-inflammatory effects in a periodontitis model by restoring the homeostasis of reactive T helper 17 (Th17) cells/Tregs [99]. Moreover, dental pulp stem cell-derived exosomes (DPSC-Exos) were also effective in treating experimental periodontitis. DPSC-Exos promoted a shift from a pro-inflammatory to an anti-inflammatory phenotype of macrophages in periodontal tissues of mice with periodontitis, and this effect may be related to miR-1246 in DPSC-Exos [100].

Although naturally derived EVs can perform important functions through a variety of biological mechanisms, they still have some limitations, such as poor targeting and insufficient numbers of effective EVs. While EVs can inherit similar properties to their parent cells, the bioactive molecules vary greatly among different EVs. Therefore, researchers are currently studying engineered EVs, which use methods to improve their performance and increase production. One approach is to genetically engineer EVs by treating parental cells before collection [101].

Genetic modification of parental cells can confer more precise functions on the vesicles they produce. Studies have shown that MSCs with up-regulated HIF-1α expression can produce exosomes that have a better effect of promoting osteogenesis, leading to significant new bone tissue regeneration [102]. Additionally, preconditioning parental cells with conditioned medium can also improve the production or bioactivity of EVs. For instance, when rat bone marrow stromal cells (rBMSCs) were pretreated with osteogenic induction medium, multiple osteogenic miRNAs were expressed in exosomes derived from rBMSCs, resulting in a 2-fold increase in alkaline phosphatase (ALP) activity compared with the blank control group [91]. Furthermore, physical operations, such as force and electrical stimulation, can alter the amount and content of derived EVs.

Exogenous engineering of EVs is also a current topic of interest. Currently, EVs can be directly modified on their surface through methods such as incubation, electroporation, ultrasonic treatment, mechanical extrusion, cyclic freezing, and thawing, as well as through covalent or non-covalent interactions [103]. By these methods, modified EVs can be loaded with desired biomolecules inside or on their surface, enhancing the function required for treatment. For example, Zha et al. used electrical pulses to create holes on the surface of EVs to increase membrane permeability and then loaded vascular endothelial growth factor (VEGF) plasmids into EVs to construct gene-activated engineering exosomes. These engineered exosomes can be used as osteogenic substrates to induce osteogenic differentiation of mesenchymal stem cells. As gene vectors, VEGF genes can be released in a controlled manner to reshape the vascular system [90].

### 4.4. Clinical Trail

The ultimate goal of these studies is to better apply them in clinical treatment. In recent years, with the continuous development of research, clinical trials related to EVs have also been gradually carried out. As of March 2024, there were 100 clinical studies with “Extracellular vesicles” as keywords, and 204 with “Exosomes” as keywords (ClinicalTrials.gov). EVs are now widely used in the treatment of various diseases, such as respiratory diseases, central nervous system diseases, infectious diseases, and more. The most studied disease is respiratory disease, which may also be affected by COVID-19. Currently, there are seven clinical trials of bone tissue-related EVs for diseases including osteoarthritis, periodontal disease, fracture, and disc disease, of which two have been completed (Table 1). Vozel et al. conducted a clinical trial in 2021 (NCT04281901) using autologous platelet- and extracellular vesicle-rich plasma (PVRP) to treat chronic postoperative temporal bone cavity inflammation, and found that the PVRP treatment group became asymptomatic faster, suggesting PVRP as a potential new treatment approach [104]. Another completed clinical trial in 2022 (NCT04849429) used platelet-rich plasma (PRP) with exosomes to treat degenerative disc disease, but no results have been published yet.

## 5. Conclusions

Extracellular vesicles are rich in biogenetic information, lipids, and a variety of proteins. Currently, researchers have been able to successfully isolate and identify extracellular vesicles from cells of different origins and apply them to the repair process of bone defects in combination with various advanced biomaterials, providing a novel therapeutic modality for promoting bone regeneration. However, there are still some questions that need to be addressed, such as whether there are differences in the effects of extracellular vesicles from different sources on bone regeneration in the same individual, the long-term efficacy of extracellular vesicles after their in vivo application, and their regression and degradation processes, and the potential for immune rejection after the injection of extracellular vesicles of cross-species origin. Much more research is required to fully explore the potential of extracellular vesicles for bone regeneration. We remain optimistic that continued study will lead to new insights and discoveries in this field.

## Figures and Tables

**Table 1:** Clinical trial of EVs for bone tissue-related diseases (ClinicalTrials.gov).

Condition or Disease	Year of Initiation	Origin	Sponsor	Status	Clinical Trial Number
Osteoarthritis	2020	Adipose-derived Stromal Cells (ASC)	Istituto Ortopedico Galeazzi, Italy	Recruiting	NCT04223622
Segmental Fracture-Bone Loss	2022	Mesenchymal stem cells enriched by extracellular vesicles	Institute of Biophysics and Cell Engineering of National Academy of Sciences of Belarus, Belarus	Not yet recruiting	NCT05520125
Otitis Media Chronic Temporal Bone	2020	Autologous blood-derived product called platelet-and extracellular vesicle-rich plasma (PVRP)	University Medical Centre Ljubljana, Slovenia	Completed	NCT04281901
Periodontitis	2020	Adipose derived stem cells exosomes	Beni-Suef University, Egypt	Recruiting	NCT04270006
Bone Loss, Alveolar	2021	Autogenous Mesenchymal Stem Cell Culture-Derived Signaling Molecules	Pontificia Universidade Católica do Rio Grande do Sul, Brazil	Not yet recruiting	NCT04998058
Osteoarthritis, Knee	2021	Exosomes derived from allogeneic mesenchymal stromal cells.	Universidad de los Andes, Chile	Not yet recruiting	NCT05060107
Degenerative Disc Disease	2021	Platelet rich plasma (PRP) with exosomes	Platelet rich plasma (PRP) with exosomes, India	Completed	NCT04849429

## Data Availability

Not applicable.
